# Interleukin-6 gene amplification and shortened survival in glioblastoma patients

**DOI:** 10.1038/sj.bjc.6603586

**Published:** 2007-01-16

**Authors:** A Tchirkov, T Khalil, E Chautard, K Mokhtari, L Véronèse, B Irthum, P Vago, J-L Kémény, P Verrelle

**Affiliations:** 1 EA 3846, Université d’Auvergne, Clermont-Ferrand, F-63001, France; 2 Service de Cytogénétique Médicale, UFR Médecine, CHU, Clermont-Ferrand, F-63001, France; 3 Departement de Radiothérapie, Centre Jean Perrin, Clermont-Ferrand, F-63011, France; 4 Services de Neurochirurgie, CHU, Clermont-Ferrand, F-63001, France; 5 Laboratoire de Neuropathologie, Groupe hospitalier Pitie-Salpetriere, Paris, F-75013, France; 6 Laboratoire d’Anatomie Pathologique, CHU, Clermont-Ferrand, F-63001, France

**Keywords:** *IL-6* gene, glioblastoma multiforme, amplification, prognosis

## Abstract

Interleukin-6 (IL-6) is known to promote tumour growth and survival. We evaluated *IL-6* gene amplification in tumours from 53 glioma patients using fluorescence *in situ* hybridisation. Amplification events were detected only in glioblastomas (15 out of 36 cases), the most malignant tumours, and were significantly associated with decreased patient survival.

## INTRODUCTION

Interleukin-6 (IL-6) is a pleiotropic cytokine that regulates the immune response, but also plays a role in promoting tumour growth and survival (Trikha *et al*, 2003; Hodge *et al*, 2005). In gliomas, the level of *IL-6* gene expression increases with the grade of malignancy (Rolhion *et al*, 2001). In glioblastoma multiforme (GBM), the most malignant glioma, amplification/overexpression of the *IL-6* gene appears to be a common feature (Tchirkov *et al*, 2001). Recent genomic array studies have reported that the number of *IL-6* gene copies was increased in 40–50% of GBM (Suzuki *et al*, 2004; Saigusa *et al*, 2005). It is however unknown whether this alteration could be found in gliomas of lower malignancy grades. The prognostic significance of *IL-6* gene amplification in GBM has not yet been determined. To address these issues, we evaluated here *IL-6* gene amplification using interphase fluorescence *in situ* hybridisation (FISH) in 53 gliomas representative of various histological types and malignancy grades. In GBM, the results were correlated with patient survival.

## MATERIALS AND METHODS

Glioma samples were taken from the material of surgical resection during the course of standard diagnostic procedure. Histological diagnosis and grading of tumours was consistent with the World Health Organization (WHO) criteria (World Health Organization, 2000). Nine tumours were classified as low-grade gliomas (grade I–II), including one pilocytic astrocytoma, three astrocytomas, two oligodendrogliomas and three oligoastrocytomas. Eight tumours were anaplastic gliomas (grade III), including six anaplastic oligoastrocytomas and two anaplastic oligodendrogliomas. The remaining 36 tumours were classified as GBM (grade IV). Patients with malignant gliomas (grade III–IV) were treated by surgery followed by either high-dose carmustine followed by radiotherapy in cases with optimal tumour resection or radiotherapy and concomitant temozolomide followed by monthly cycles of adjuvant temozolomide in cases with partial tumour resection or biopsy alone.

An interphase FISH was performed on frozen sections of gliomas mounted on Fisher Superfrost slides. The *IL-6* gene probe was designed from the bacterial artificial chromosome clone RP11-240H8 (GenBank Accession Number AC073072) kindly provided by Professor Mariano Rocchi (University of Bari, Italy). The *IL-6* probe labelled with SpectrumGreen (Vysis/Abbott, Rungis, France) and chromosome 7 cetromeric probe labelled with SpectrumOrange (Vysis/Abbott) were co-hybridised to evaluate simultaneously the number of *IL-6* gene and chromosome 7 copies. A median of 148 nuclei (116–245, interquartile range) was scored using a Metafer4-MetaCyte microscope scanning system (MetaSystems, Le Cannet, France).

The number of *IL-6* mRNA transcripts was assessed using quantitative real-time reverse transcriptase-PCR (qRT–PCR) in the LightCycler system (Roche Diagnostics, Meylan, France) with *IL-6* specific primers (Tchirkov *et al*, 2001) and normalised to the expression of a housekeeping gene, *ABL* (Tchirkov *et al*, 2003).

Analyses of statistical links between biological and clinical characteristics were performed using standard tests. Overall survival was calculated using the Kaplan–Meier method and survival curves were compared using the log-rank test. A multivariate analysis was performed using the Cox regression model.

## RESULTS

**Figure 1:**
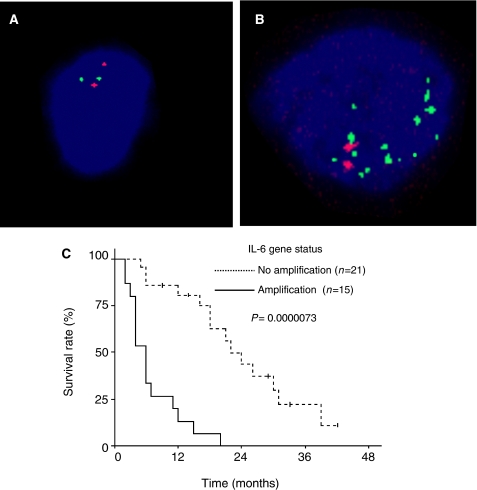
(**A** and **B**) Example of FISH analysis of chromosome 7 (red signals) and *IL-6* gene (green signals) copy numbers in frozen sections of glioma tumours. A nucleus showing two copies of chromosome 7 and two copies of *IL-6* gene (**A**); a nucleus with two copies of chromosome 7 and a high-level amplification of *IL-6* gene (ratio of *IL-6* to centromere 7 signals greater than 3) (**B**). (**C**) Overall survival as a function of *IL-6* gene status in GBM patients. Patients with *IL-6* gene amplification had significantly shorter survival than patients without amplification.

Using interphase FISH approach, no *IL-6* gene amplification was detected in low-grade or anaplastic tumours (*n*=17), whereas high-level amplification was found in 15 out of 36 (41.7%) GBM (Figure 1A and B). The percentage of nuclei with amplification events varied between 21 and 77% (33%, median).

Quantification of *IL-6* mRNA with qRT–PCR revealed a highly significant increase in the mean *IL-6*/*ABL* ratio in GBM (33.3%) as compared with non-GBM (1.5%; Kruskal–Wallis test, *P*=3.8 × 10^−6^). The mean *IL-6*/*ABL* ratio was >10-fold greater in GBM manifesting *IL-6* gene amplification than in GBM without amplification (71.6 *vs* 6.1%; *P*=4.3 × 10^−7^).

GBM patients with amplified *IL-6* gene had significantly shorter survival than patients without amplification (log-rank test, *P*=0.0000073; Figure 1C). Multivariate Cox analysis for overall survival, including *IL-6* amplification, extent of tumour resection and age as variables, demonstrated that *IL-6* amplification was an independent factor of poor prognosis (relative risk (RR) amplified *vs* non-amplified 8.07; *P*=0.000016). The complete tumour resection, equally distributed among patients with and without *IL-6* gene amplification, was an independent factor of favourable prognosis (RR complete *vs* partial 0.31; *P*=0.013). Age did not influence survival of patients.

## DISCUSSION

Glioblastomas are the most devastating primary brain tumours. Despite modern treatments, about 40% of patients with GBM die within 6 months after diagnosis (Reardon *et al*, 2006). In the present study, we demonstrated that one of the molecular abnormalities associated with the aggressiveness of GBM is the amplification of the *IL-6* gene, which was found in 41.7% of patients and significantly correlated with decreased survival.

Amplifications on chromosome 7p in GBM are believed to be driven by the amplification of the epidermal growth factor receptor (EGFR) gene locus (Rossi *et al*, 2005). This initial amplification event may induce instability along the length of this chromosomal arm, resulting in co-amplification of other genes. Of note, *EGFR* gene amplification has no clear prognostic value in GBM (Houillier *et al*, 2006). It may be possible that some of co-amplified (or independently amplified) genes are involved in glioma development and progression and are important for patient prognosis. Novel amplicons on chromosome 7p distinct from *EGFR* were recently identified using high-resolution genomic analyses and were reported to contain, among other genes, the *IL-6* gene that was overexpressed (Rossi *et al*, 2005).

In gliomas, IL-6 plays a role in promoting tumour growth (Goswami *et al*, 1998) and angiogenesis (Loeffler *et al*, 2005). This cytokine also protects cancer cells from apoptotic depletion during chemotherapy and radiotherapy through activation of the Janus kinase/STAT and phosphatidylinositol 3-kinase/AKT pathways (Miyamoto *et al*, 2001; Trikha *et al*, 2003; Hodge *et al*, 2005). In this context, amplification of the *IL-6* gene leading to its overexpression is likely one of the major factors contributing to the aggressiveness and poor response to therapies of GBM. It may therefore be suggested that targeting IL-6 and its signalling pathways in GBM would sensitise these otherwise resistant tumours to chemotherapeutic drugs and radiotherapy.
